# Multiple Primary Malignancies With Hypercalcemia Presentation: A Case Report

**DOI:** 10.7759/cureus.24266

**Published:** 2022-04-19

**Authors:** Sushmitha Diraviam, Asseel Al-Bayati, Oksana Mayovska, Rabhea Chaudhry, Swapnil V Patel

**Affiliations:** 1 Internal Medicine, St. George’s University, West Indies, GRD; 2 Internal Medicine, Jersey Shore University Medical Center, Neptune City, USA; 3 Internal Medicine, University of Baghdad College of Medicine, Baghdad, IRQ; 4 Internal Medicine, Morristown Medical Center, Morristown, USA

**Keywords:** paraneoplastic hypercalcemia, colorectal cancer, rectal adenocarcinoma, lung squamous cell carcinoma, multiple primary cancers

## Abstract

Multiple primary malignancies (MPMs) are defined as having more than one primary malignancy and when each tumor is histologically distinct and unrelated to the others. Multiple risk factors have been found to be associated with MPMs. These include familial syndromes, sequela from treatments of previous malignancies, and environmental factors such as smoking, alcohol consumption, obesity, and male sex. Hypercalcemia has a well-known association with malignancy and is often the first abnormality that leads to further testing. Lung cancer followed by colorectal cancer has the highest mortality of all cancers in the USA, with adenocarcinoma being the most prevalent histological subtype. Further, estimates show that those with one malignancy have a 1.29 times higher risk of developing another malignancy.

Hereby, we present a case of a patient with squamous cell carcinoma of the lung who presented with hypercalcemia and incidentally was found to have another primary adenocarcinoma of the colon.

## Introduction

The incidence of multiple primary malignancies (MPMs) varies from 2.4% to 17% and has been increasing due to advances in cancer research, the survival of patients with cancer, and improvements in medical imaging [1]. Factors that increase the risk of multiple malignancies include familial syndromes, hormonal and environmental factors, and treatment of previous malignancies [2]. Lung cancer, in particular, has the highest mortality of all cancers in the USA, with non-small cell lung cancer (NSCLC) comprising 85% of diagnoses and squamous cell carcinoma comprising 30% of all NSCLCs as the second most common type of lung cancer following lung adenocarcinoma [3]. Fortunately, the incidence has been decreasing with a reduction in tobacco consumption [4].

On the other hand, cancers of the colon and rectum are the third most common cancer worldwide, and incidence has been increasing especially in high-income, industrialized countries. The majority of cases occur sporadically, and diet is the most commonly cited extrinsic risk factor. The most common histological subtype is adenocarcinoma, which accounts for 90%-95% of all large bowel tumors [5].

Hypercalcemia as a paraneoplastic syndrome has been reported with both squamous cell carcinoma of the lung and colorectal adenocarcinoma, more commonly occurring with the former malignancy. However, it has been documented to be a poor prognostic factor for colorectal cancer [6].

## Case presentation

**Figure 1:**
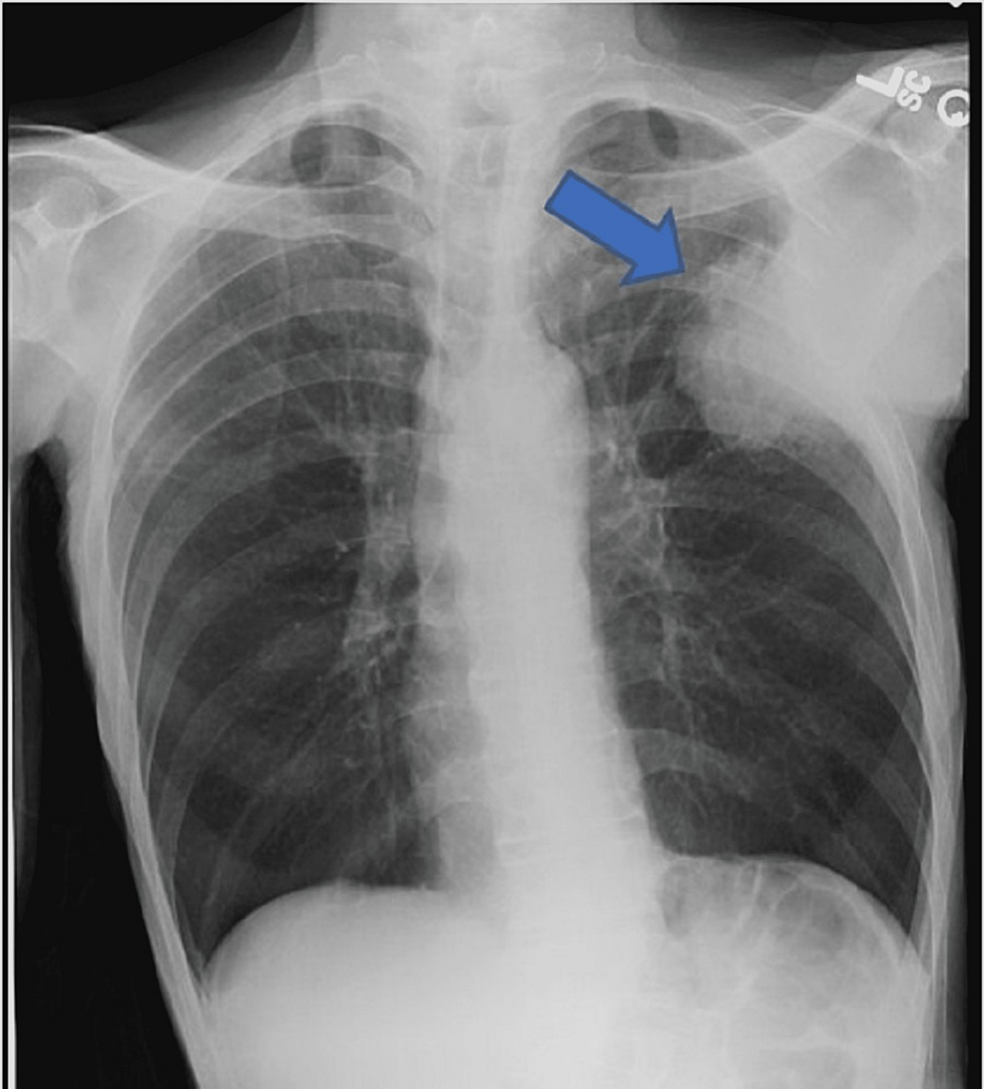
CXR showing left chest wall mass (arrow), with erosions of the third and fourth ribs. The mass measures at least 7 × 8.5 cm in size as it projects into the left lung. The right lung remains clear. The cardiac and mediastinal contours appear normal.

**Figure 2:**
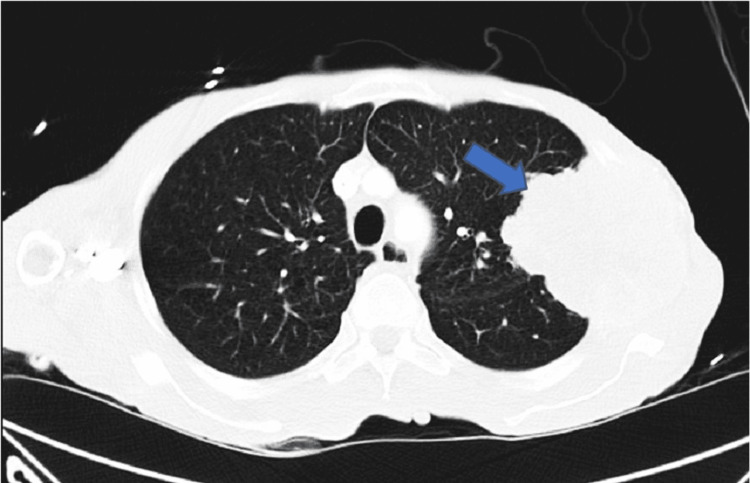
Chest CT showing a large lobulated mass (arrow) with partially spiculated margins medially, estimated at 8.4 × 9.5 × 9.7 cm in its greatest dimension. It demonstrates heterogeneous enhancement with peripheral neovascularization. It has invaded through the upper left chest wall with destruction of the left second to fourth ribs. It is abutting the superior aspect of the fifth rib.

**Figure 3:**
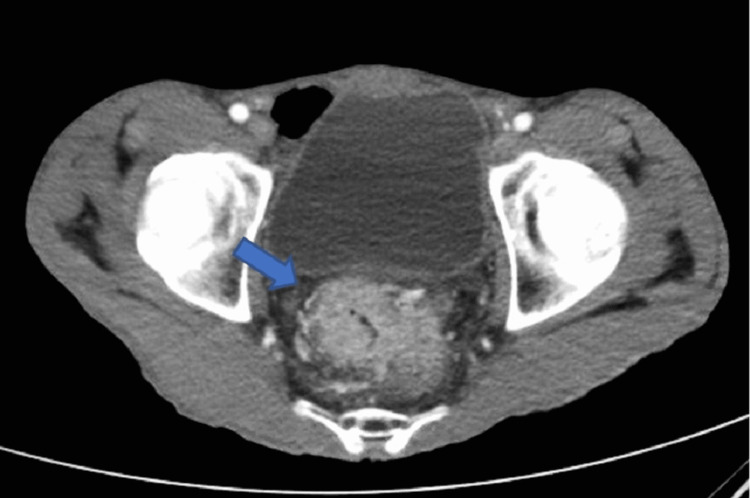
Circumferential wall enhancement of the mid-rectal segment, suspicious for an “apple core” neoplastic process (arrow). The inferior aspect is located at approximately 6.5 cm from the anal verge. This disease segment measures 7.2 cm in length. Neovascularization is noted.

**Figure 4:**
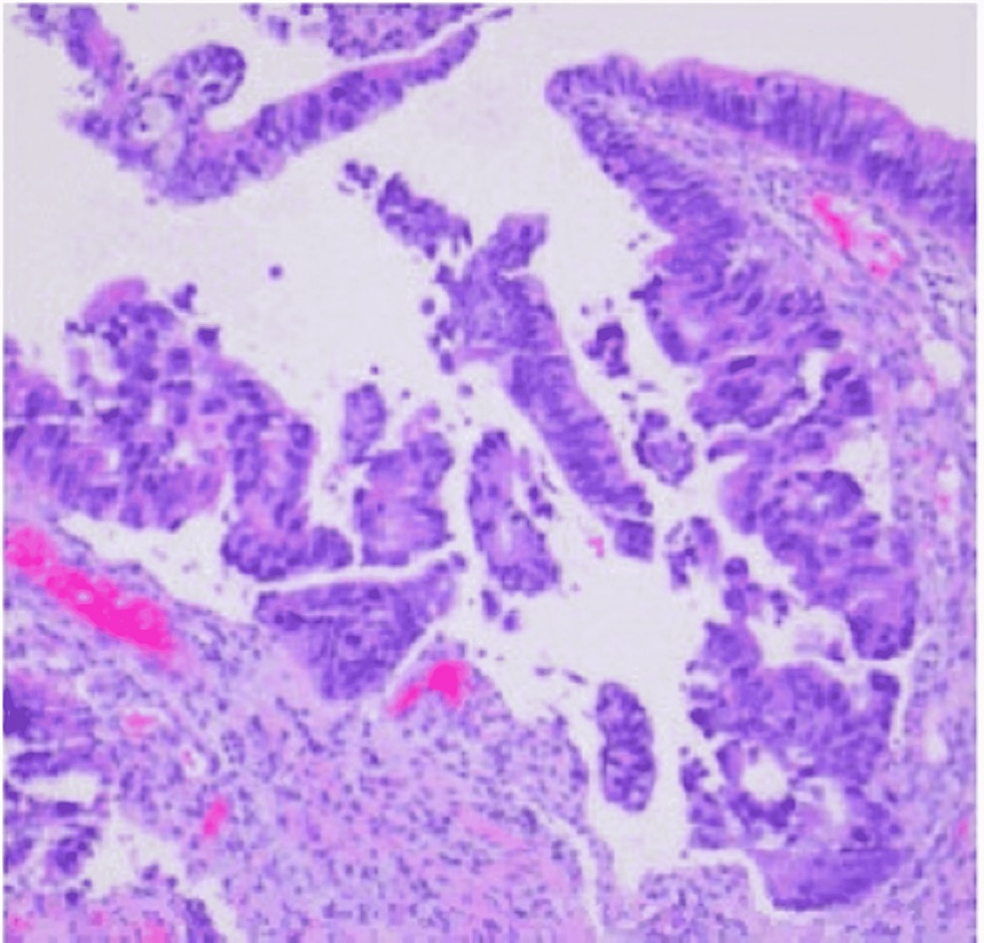
Rectal mass biopsy showing superficial mucosal tissue lined by highly atypical dysplastic/neoplastic glandular epithelial cells with nuclear pleomorphism, prominent nucleoli, and frequent mitotic figures. This is consistent with at least intramucosal adenocarcinoma. Invasive growth cannot be excluded.

A 58-year-old African American male with a history of tobacco use disorder and paranoid-type schizophrenia presented to the emergency department due to progressively worsening chest pain for six months. He complained of chest pain that was 9/10 in intensity, sharp, and constant, and radiated to his throat. He denied aggravation of pain with positional changes or in relation to meals. The chest pain was associated with intermittent shortness of breath, but the patient denied associated diaphoresis, nausea, fever, or coughing. A review of systems was positive for chronic diarrhea, malaise, and weight loss, but the patient was unable to quantify the amount of weight loss. Physical examination revealed a cachectic man with a BMI of 15 kg/m^2^, blood pressure of 122/70 mmHg, and pulse of 122 bpm. Examination of the respiratory system showed decreased breath sounds in the left upper and middle lung fields, dullness to percussion on the left upper and middle lung fields, and reproducible chest pain upon palpation. Laboratory investigations are shown in Table 1. Chest X-ray (CXR) (Figure 1) displayed a left chest wall mass with erosions of the third and fourth ribs. The patient was started on IV fluids and zoledronic acid and calcitonin for hypercalcemia. A follow-up CT scan of the chest (Figure 2) revealed a large left upper lobe and a neoplastic process invading adjacent structures. CT of the abdomen/pelvis (Figure 3) showed an enhancing rectal mass, highly suspicious for a neoplastic process, with an “apple core” appearance. CT scan also showed scattered findings of a tiny left adrenal nodule, subcentimeter hepatic lesions, and osseous findings suspicious for metastasis. Subsequently, CT-guided needle core biopsy of the lung tumor with immunohistochemical stains yielded positive for p40, CK 5/6, and PD-1 and negative for TTF-1 and napsin A, supporting the diagnosis of stage IV squamous cell carcinoma with erosion to the bone. Colonoscopy with rectal mass biopsy confirmed abnormal growth of superficial mucosal tissue lined by highly atypical dysplastic/neoplastic glandular epithelial cells showing nuclear pleomorphism, prominent nucleoli, and frequent mitotic figures. This is consistent with intramucosal adenocarcinoma (Figure 4). Those biopsy results confirmed two separate primary malignancies.

The patient was deemed to be a poor surgical candidate and received palliative radiation for pain control, which he tolerated well. While admitted, the patient developed episodes of asymptomatic nocturnal sinus pauses, which were thought to be related to cancer. A temporary transvenous pacemaker was implanted, which was monitored in the ICU. Over the next several weeks, the patient’s condition continued to deteriorate in the medical wards with complications including pleural effusion, mastoid effusion, delirium, and recurrent fevers secondary to the neoplastic process. Eventually, a family meeting was held to discuss goals of care, and the decision to transition to outpatient hospice care was made.

**Table 1:** Laboratory results

Test	Result	Reference range
Troponin	0.01 ng/mL	<0.04 ng/mL
Sodium	134 mmol/L	136-145 mmol/L
Potassium	4.1 mmol/L	3.5-5.2 mmol/L
Chloride	97 mmol/L	96-110 mmol/L
Glucose	101 mg/dL	70-99 mg/dL
Calcium	14.4 mg/dL	8.5-10.5 mg/dL
Creatinine	1.31 mg/dL	0.61-1.24 mg/dL
Hemoglobin	12.3 g/dL	12-17.5 g/dL
Mean corpuscular volume (MCV)	85.5 fL	80-100 fL
WBC	12.9 × 10^3^/uL	4.5-11 × 10^3^/uL
Parathyroid hormone-related peptide (PTHrP)	35 pg/mL	<2.5 pg/mL
Parathyroid hormone (PTH)	12.2 ng/mL	14-65 pg/mL

## Discussion

Multiple primary malignancies (MPMs) have been reported frequently in the literature. However, it remains a rare condition with an incidence rate of 2.4%-17% [1]. The Surveillance, Epidemiology, and End Results (SEER) Program and the International Association of Cancer Registries and International Agency for Research on Cancer (IACR/IARC) agreed on defining MPMs as the diagnosis of more than one cancer in the same patient [1]. The diagnosis of MPMs must meet three characteristic criteria: (I) each tumor must be distinct from each other, (II) each tumor must present definite features of malignancy, and (III) the possibility that the one is a metastasis of another must be ruled out [7,8].

They are divided into two major categories. They are called synchronous if the tumors were diagnosed simultaneously at the same time or within a six-month interval. If the interval is longer, then they are called metachronous [7,8].

The advancement of the detection and diagnosis of cancers helped recognize cancers in their early stages; however, progress is still underway to define the risk factors and etiology behind the development of multiple neoplasms in the same patient. To find the incidence of second and third malignancy, Schoenberg estimated that patients with cancer had 1.29 times the risk of developing new malignancy compared with those who were never diagnosed with cancer before [9]. Generally, the etiology of MPMs can be divided into three major groups. The first group includes treatment-related risk factors, the second includes syndromic conditions, and the third includes cancer with common etiologic risk factors, such as genetic predisposition or environmental factors [10]. Host and lifestyle-related factors contribute to the causes of multiple primary cancers. Similar to our reported case, it was also found that men have a higher risk for synchronous lesions compared to women who tend to have metachronous MPMs [11]. Smoking, alcohol consumption, and obesity have also been found to have significant temporal changes in the prevalence of cancer risk [1].

Paraneoplastic hypercalcemia carries a very poor prognosis regardless of the mechanism. It is estimated that 50% of patients who present with paraneoplastic hypercalcemia die within 30 days [12]. Hypercalcemia in malignancy can be secondary to local osteolytic hypercalcemia, humoral hypercalcemia of malignancy (parathyroid hormone-related peptide (PTHrP)), 1,25 (OH)2 D-secreting lymphomas that enhances renal calcium retention, or ectopic hyperparathyroidism [13].

Our reported case seems to share multiple risk factors predisposing him to multiple cancers; some of them were modifiable, such as obesity and smoking. However, the aggressive nature of cancer and the association with paraneoplastic hypercalcemia posed a clinical challenge and burden to the patient and his family.

## Conclusions

Above all, multiple primary malignancies cause a lot of trouble to both clinicians and patients as they limit therapeutic options and the quality of life. We are hopeful that reports similar to this will be useful in developing effective screening and surveillance protocols, aiming to early detect and treat patients effectively with a better outcome.

## References

[1] Alberg AJ, Brock MV, Ford JG, Samet JM, Spivack SD (2013). Epidemiology of lung cancer: diagnosis and management of lung cancer, 3rd ed: American College of Chest Physicians evidence-based clinical practice guidelines. Chest.

[2] Aydiner A, Karadeniz A, Uygun K, Tas S, Tas F, Disci R, Topuz E (2000). Multiple primary neoplasms at a single institution: differences between synchronous and metachronous neoplasms. Am J Clin Oncol.

[3] Copur MS, Manapuram S (2019). Multiple primary tumors over a lifetime. Oncology (Williston Park).

[4] Derwinger K, Gustavsson B (2011). A study of aspects on gender and prognosis in synchronous colorectal cancer. Clin Med Insights Oncol.

[5] Kobayashi H, Gieniec KA, Lannagan TR (2022). The origin and contribution of cancer-associated fibroblasts in colorectal carcinogenesis. Gastroenterology.

[6] Heist RS, Sequist LV, Engelman JA (2012). Genetic changes in squamous cell lung cancer: a review. J Thorac Oncol.

[7] Labianca R, Beretta GD, Kildani B (2010). Colon cancer. Crit Rev Oncol Hematol.

[8] Ralston SH, Gallacher SJ, Patel U, Campbell J, Boyle IT (1990). Cancer-associated hypercalcemia: morbidity and mortality. Clinical experience in 126 treated patients. Ann Intern Med.

[9] Schoenberg BS (1977). Multiple primary malignant neoplasms. The Connecticut experience, 1935-1964. Recent Results Cancer Res.

[10] Soerjomataram I, Coebergh JW (2009). Epidemiology of multiple primary cancers. Methods Mol Biol.

[11] Spratt JS Jr, Hoag MG (1966). Incidence of multiple primary cancers per man-year of follow up: 20-year review from the Ellis Fischel State Cancer Hospital. Ann Surg.

[12] Stewart AF (2005). Clinical practice. Hypercalcemia associated with cancer. N Engl J Med.

[13] Takalkar U, Asegaonkar BN, Kodlikeri P, Asegaonkar S, Sharma B, Advani SH (2013). An elderly woman with triple primary metachronous malignancy: a case report and review of literature. Int J Surg Case Rep.

